# Supplementary material for: Conserved role of spike S2 domain N-glycosylation across betacoronaviruses
Source: Npj Viruses. 2025 Jan 25;3:4. doi: 10.1038/s44298-024-00085-7 (PMC11762317; doi:10.1038/s44298-024-00085-7)
Supplement: Supplementary file 1 — SupplementaryMaterial_sub [file 44298_2024_85_MOESM1_ESM.pdf]

Supplementary Material

Conserved role of Spike S2 domain N-glycosylation across *betacoronaviruses*

Yang *et al.*

Contents:

Supplementary Videos S1-S5
Supplementary Table S1
Supplementary Figures S1-S5

Supplementary Video legends:

Video S1: Syncytia formation: parent spike over 24h
Video S2: Syncytia formation: G1 over 24h
Video S3: Syncytia formation: G1345 over 24h
Video S4: Syncytia formation: G12345 over 24h
Video S5: Syncytia formation: no spike over 24h

Table S1
Yang *et al.*

Run 1:

	similarity score (n=1000 cells being analyzed)					
sample	FITC/Spike vs. Alexa647/GM130/Cis-Golgi	FITC/Spike vs. Alexa555/CANX/ER	FITC/Spike vs. Alexa405/ WGA/membrane	Alexa647/GM130/Cis-Golgi vs. Alexa555/ CANX/ER	Alexa647/GM130/Cis-Golgi vs. Alexa405/ WGA/membrane	Alexa555/CANX/ER vs. Alexa405/ WGA/membrane
parent	0.1564±0.5611	0.9496±0.891	-0.108±0.5222	0.2067±0.5877	-0.24±0.5322	-0.2265±0.5191
G1	0.2211±0.646	1.451±0.9696	-0.1444±0.5611	0.1395±0.6188	-0.159±0.6318	-0.1513±0.5605
G12	0.1732±0.5908	1.531±0.9687	-0.1991±0.5252	0.1133±0.6231	-0.2062±0.5578	-0.1912±0.5545
G13	0.15±0.6197	1.179±1.074	-0.1292±0.5735	0.1996±0.6142	-0.2028±0.5281	-0.1669±0.5882
G14	0.1447±0.6106	1.246±1.02	-0.1302±0.5452	0.1565±0.5702	-0.1809±0.5734	-0.1431±0.5506
G15	0.1864±0.6496	1.094±1.102	-0.08365±0.569	0.2019±0.6435	-0.2235±0.5782	-0.1783±0.5832
G145	0.1957±0.6407	1.278±1.057	-0.1005±0.547	0.1816±0.6248	-0.1259±0.5878	-0.1223±0.568
G1345	0.248±0.6941	1.303±1.046	-0.1375±0.5343	0.1983±0.6155	-0.09524±0.6266	-0.1152±0.5671
G12345	0.3058±0.6659	1.538±0.9504	-0.1539±0.5794	0.184±0.637	-0.08619±0.6765	-0.1251±0.5941

Run 2:

	similarity score (n=1000 cells being analyzed)					
sample	FITC/Spike vs. Alexa647/GM130/Cis-Golgi	FITC/Spike vs. Alexa555/CANX/ER	FITC/Spike vs. Alexa405/ WGA/membrane	Alexa647/GM130/Cis-Golgi vs. Alexa555/ CANX/ER	Alexa647/GM130/Cis-Golgi vs. Alexa405/ WGA/membrane	Alexa555/CANX/ER vs. Alexa405/ WGA/membrane
parent	0.1791±0.5658	0.904±0.8646	-0.09005±0.533	0.1695±0.5479	0.007704±0.6058	-0.09598±0.5402
G1	0.2041±0.597	1.255±0.944	-0.1852±0.5313	0.2308±0.5774	-0.154±0.5261	-0.1876±0.522
G12	0.1894±0.6484	1.282±0.8817	-0.1609±0.5259	0.213±0.579	-0.05477±0.6151	-0.1405±0.5512
G13	0.1709±0.6578	1.244±0.985	-0.199±0.5273	0.2054±0.5902	0.01623±0.6213	-0.1528±0.5639
G14	0.1866±0.6527	1.176±1.014	-0.1556±0.4974	0.1974±0.5654	-0.08994±0.5674	-0.1232±0.51
G15	0.2165±0.6435	1.329±0.9984	-0.1733±0.5087	0.2283±0.5972	-0.1441±0.5972	-0.1926±0.5062
G145	0.2188±0.6484	1.118±1.037	-0.1525±0.5038	0.2465±0.6204	-0.1018±0.5774	-0.156±0.56
G1345	0.3233±0.6574	1.395±0.9685	-0.1597±0.5372	0.2702±0.5882	-0.09847±0.5686	-0.1831±0.5161
G12345	0.2279±0.6416	1.326±0.9871	-0.2226±0.4978	0.2622±0.5696	-0.04683±0.602	-0.2019±0.522

Run 3:

	similarity score (n=5000 cells being analyzed)					
sample	FITC/Spike vs. Alexa647/GM130/Cis-Golgi	FITC/Spike vs. Alexa555/CANX/ER	FITC/Spike vs. Alexa405/ WGA/membrane	Alexa647/GM130/Cis-Golgi vs. Alexa555/ CANX/ER	Alexa647/GM130/Cis-Golgi vs. Alexa405/ WGA/membrane	Alexa555/CANX/ER vs. Alexa405/ WGA/membrane
parent	0.196±0.5787	0.977±0.8283	-0.203±0.4811	0.1469±0.5386	-0.1175±0.5192	-0.2754±0.5062
G1	0.1931±0.5928	1.101±0.8682	-0.1987±0.4794	0.1646±0.5646	-0.1743±0.4936	-0.2772±0.4785
G12	0.1798±0.5916	1.096±0.8531	-0.2009±0.4797	0.1269±0.5523	-0.1357±0.4984	-0.2788±0.4885
G13	0.1985±0.5851	1.09±0.8742	-0.1991±0.4701	0.1557±0.5451	-0.1603±0.5092	-0.2778±0.4776
G14	0.2002±0.5961	1.028±0.8339	-0.1992±0.4738	0.1606±0.5519	0.1329±0.5092	-0.263±0.4976
G15	0.2293±0.5855	1.109±0.9019	-0.1601±0.4912	0.1437±0.5446	-0.1184±0.5085	-0.2467±0.4966
G145	0.1988±0.5577	1.115±0.9047	-0.1858±0.4654	0.152±0.5464	-0.1781±0.4863	-0.2483±0.477
G1345	0.1937±0.5912	1.094±0.8358	-0.2457±0.4672	0.1694±0.5554	-0.1404±0.5026	-0.3028±0.4782
G12345	0.2173±0.6135	1.167±0.8658	-0.2469±0.4738	0.1864±0.5637	-0.1715±0.4798	-0.3097±0.4783

Supplementary Table S1 legend (related to Figure 1). Similarity scores showing co-localization between: **i)** spike and cis-Golgi (GM130), **i)** spike and ER (CANX), **iii)** spike and cell membrane (WGA), **iv)** cis-Golgi (GM130) and ER (CANX) markers, **v)** cis-Golgi (GM2130) and cell membrane (WGA) markers, and **vi)** ER (CANX) and cell membrane (WGA) markers. This analysis is presented for different stem S2 N-glycan mutants in three independent runs (Run 1-3). Strongest correlation was observed between spike and ER in all cases. Upon knocking out the stem N-glycans, similarity score increased for spike co-localization with ER and cis-Golgi markers, suggesting increased retention in intracellular compartments (see data in red box).

Figure S1
Yang *et al.*

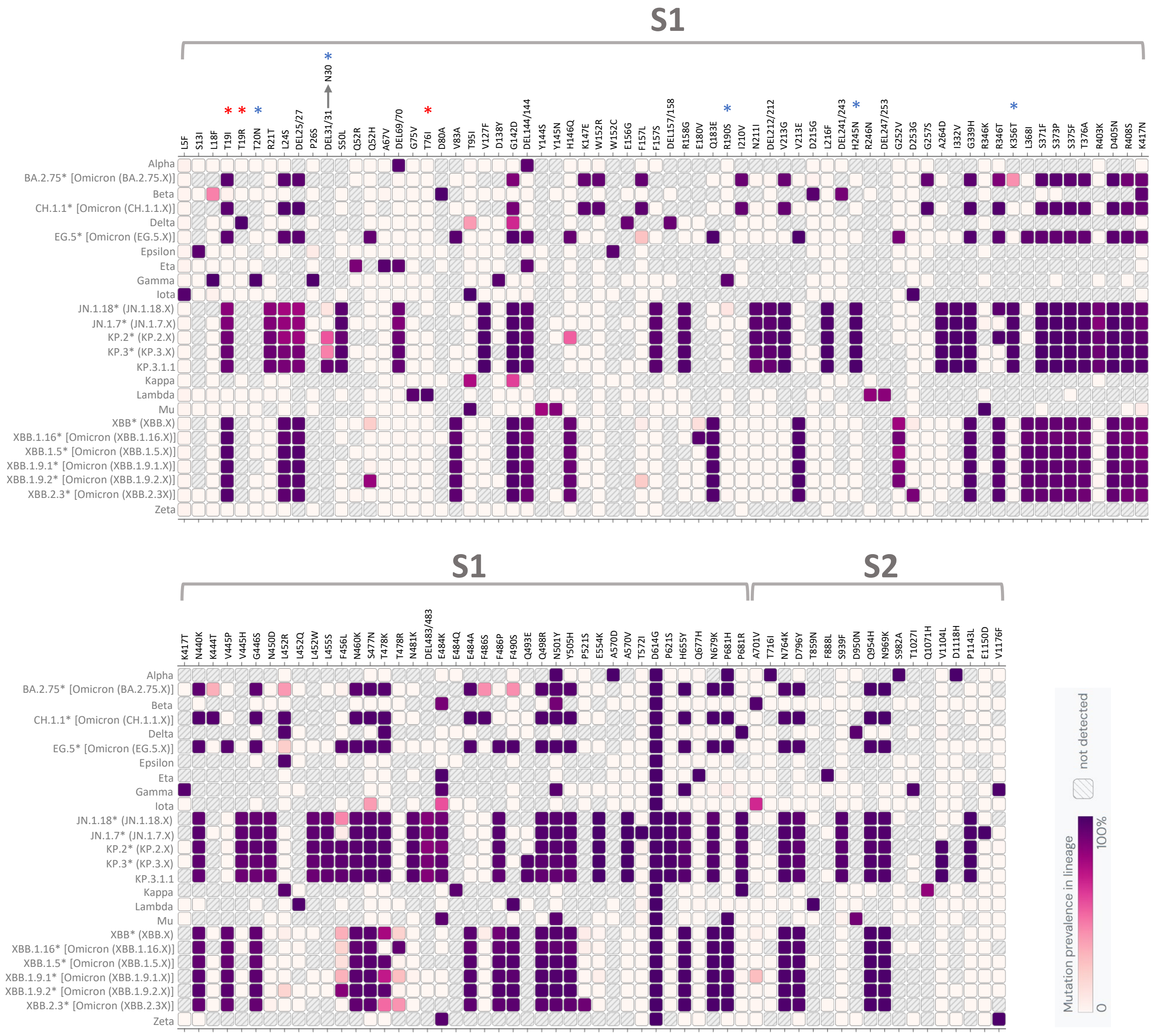

Figure S1. Mutation prevalence across SARS-CoV-2 lineages (Related to Figure 1). Figure shows amino acid mutations detected on SARS-CoV-2 spike in different W.H.O. (World Health Organization) variants of interest or concern. Red asterisk highlights glycosylation sites lost by individual viral strains and blue asterisk highlighting the new glycosylation sites that have appeared. Note that deletion of S31 results in a new glycan gained at N30. In general, many more mutations are observed in S1 region compared to spike S2, while N-glycosylation mutations only occurred in S1. Data are rendered using dashboard at outbreak.info, using GISAID data. Only mutations with >75% prevalence in a single lineage are plotted. Each lineage is sequenced at least 1000 times.

Figure S2
Yang *et al.*

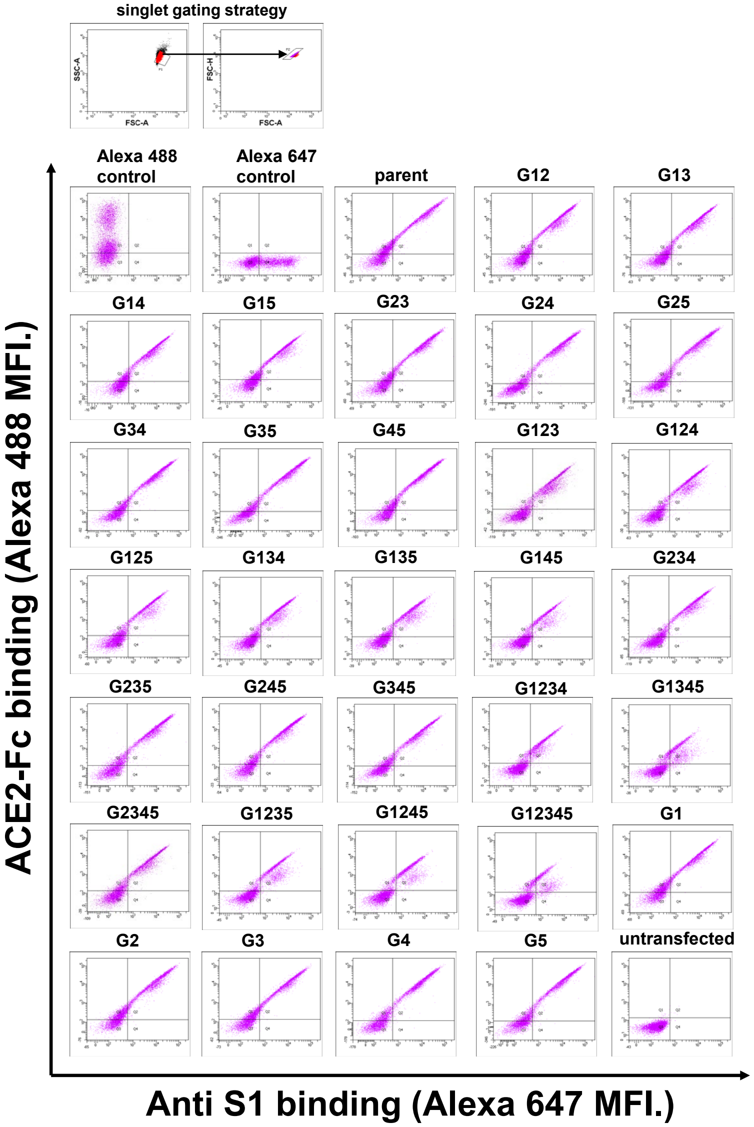

Figure S2. Effects of S2 stem N-glycans on spike protein (Related to Figure 1). Representative flow cytometry dot plot showing raw data used to generate **Figure 1b**. A direct correlation is observed between anti-S1 and ACE2-Fc binding in all cases. Single color controls are presented to demonstrate proper cytometer compensation settings.

Figure S3
Yang *et al.*

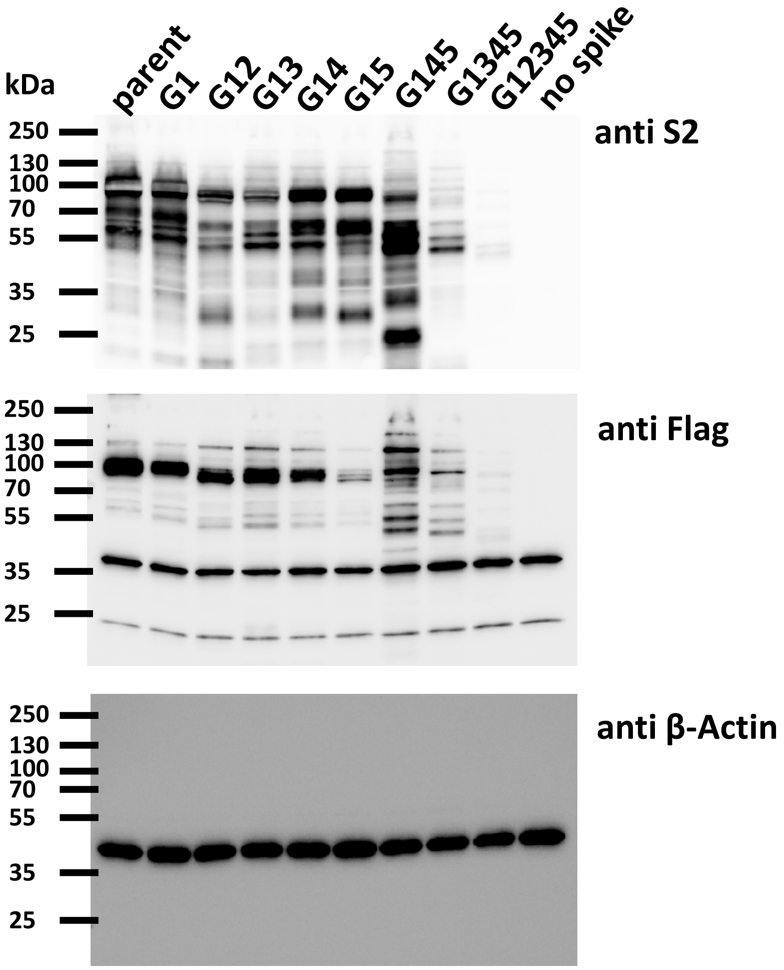

Figure S3 (Related to Figure 1). Full blots of **Figure 1d**.

Figure S4
Yang *et al.*

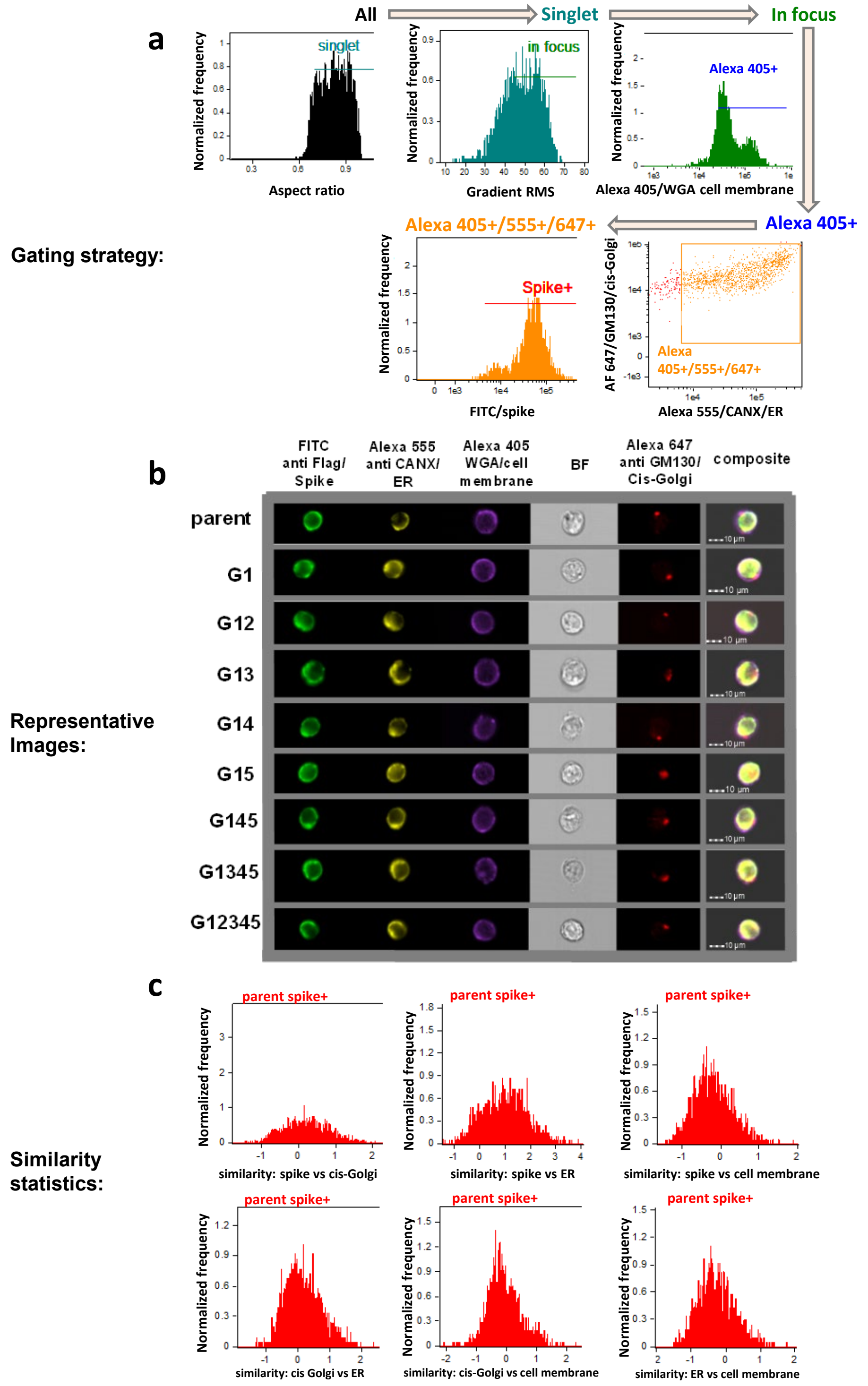

Figure S4. Image cytometry studies of spike localization (Related to Figure 1). a) Gating strategy for imaging cytometry: Aspect ratio was used for gating 'singlets', and Gradient RMS for 'in focus' cells. Four fluorescence channels were subsequently used for gating quadruple-positive cell populations. **b)** Representative fluorescence images showing FITC-anti-Flag, Alexa 555-anti CANX, Alexa 647-anti GM130 and Alexa 405-wheat germ agglutinin (WGA) used for staining spike protein, endoplasmic reticulum (ER), cis-Golgi and cell membrane, respectively. **c)** Representative similarity statistics analysis histograms. The strongest correlation was observed between spike vs ER.

Figure S5
Yang *et al.*

N=1
(main)

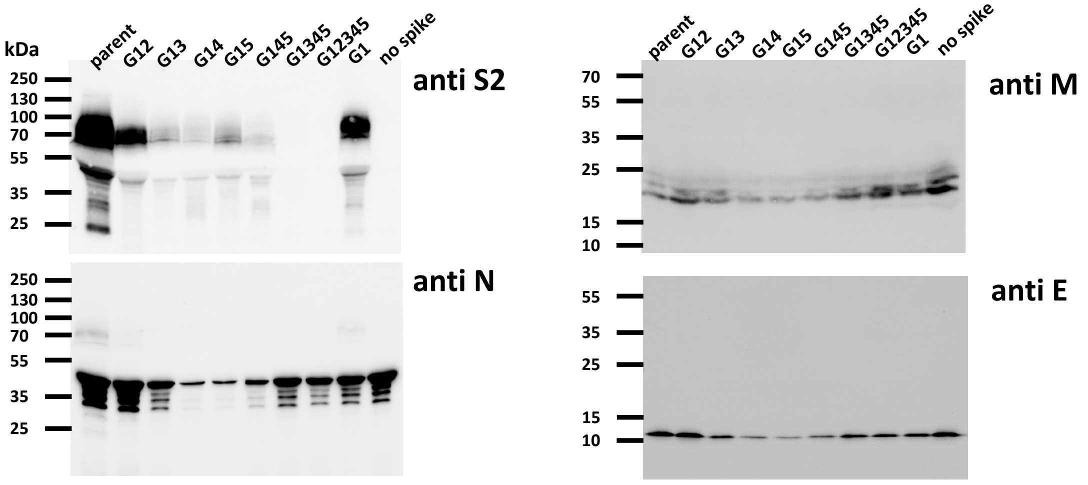

N=2

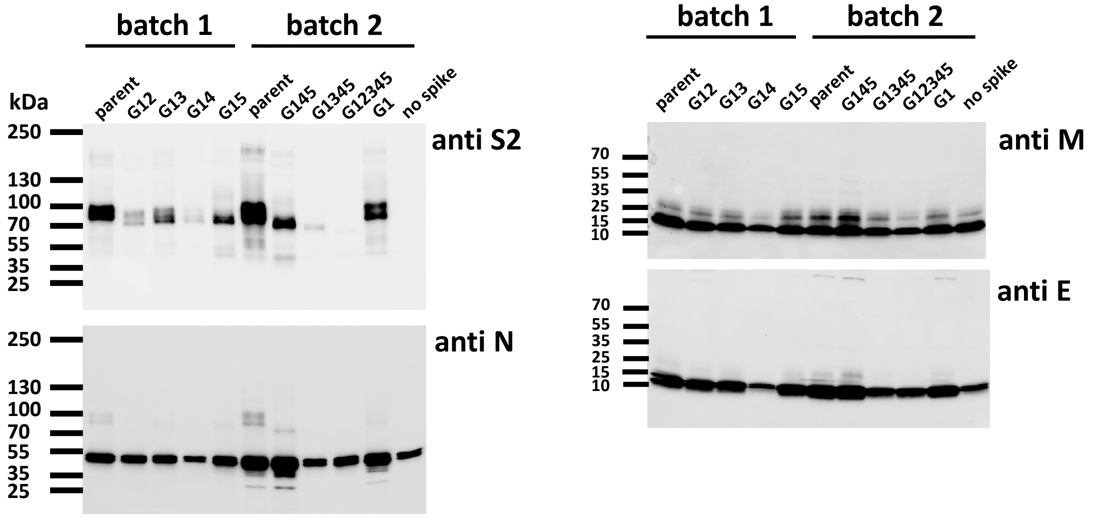

Figure S5 (Related to Figure 3). Full blots of **Figure 3c** with an additional biological repeats. Spike incorporation into VLPs was reduced upon stem glycan deletion.

Figure S6
Yang *et al.*

N=1 (main)

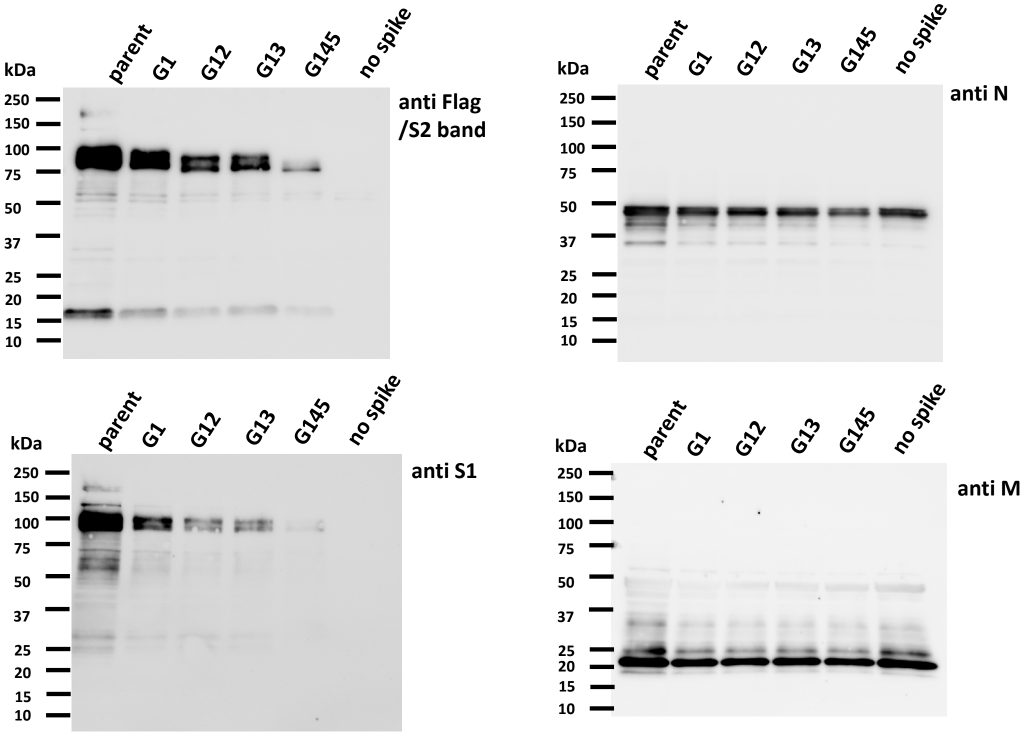

N=2

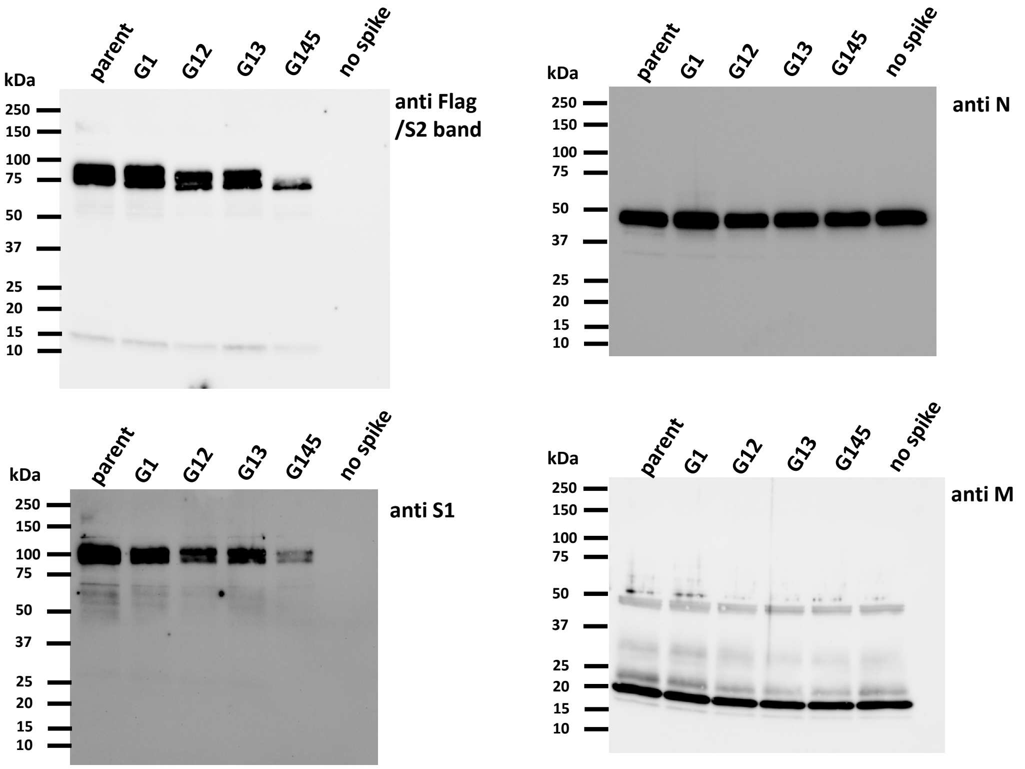

Figure S6
Yang *et al.*

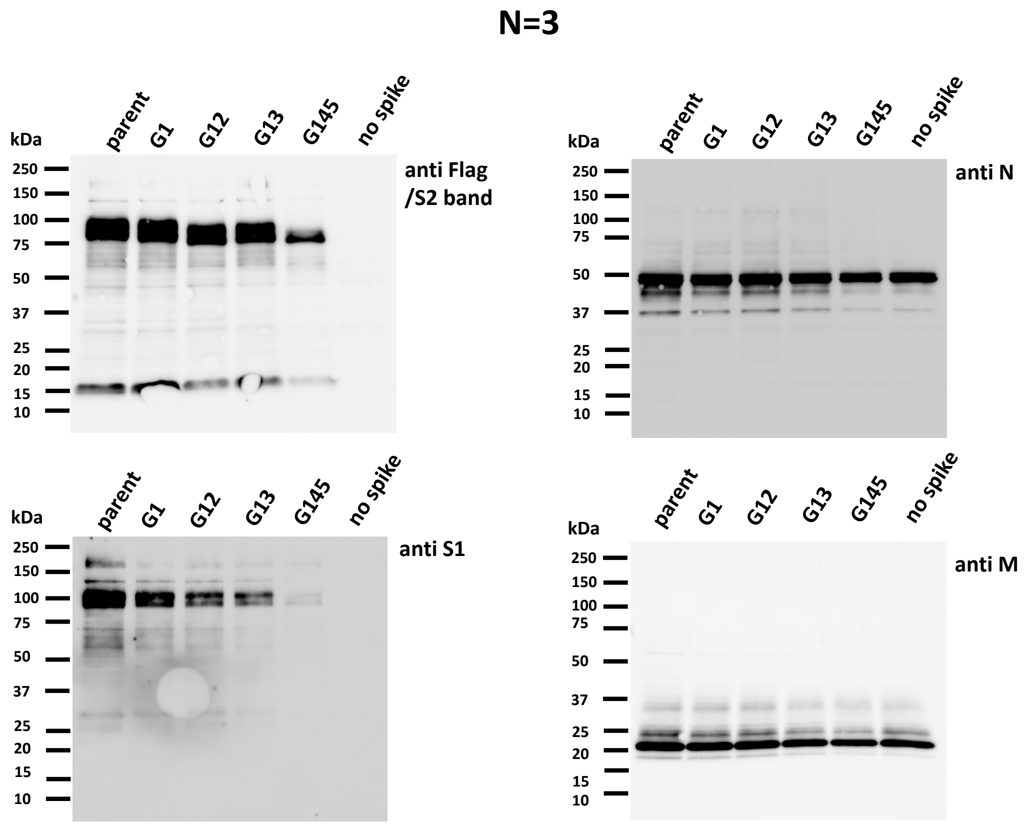

Figure S6 (Related to Figure 3). Full blots of main **Figure 3d** along with two additional biological repeats for experiment in **Figure 3d** were shown. Results demonstrated that S1 shedding was increased upon implementing stem glycan mutations.

Figure S7
Yang *et al.*

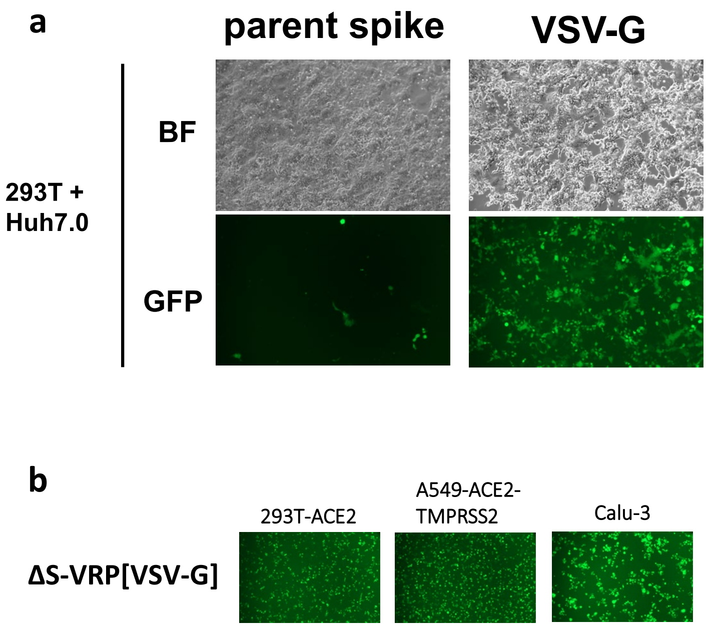

Figure S7. Replicon synthesis (Related to Figure 4). a) Fluorescence images, 72 h post-transfection, of 293T plus Huh7.0 producer cells used for making SARS-CoV-2 ∆S-VRPs complemented with spike or VSV-G. Parent spike producer cells showed limited signal but VSV-G more efficiently transmitted virus among cells thus enhancing VRP production. **b)** Three type of target cells were infected by ∆S-VRP[VSV-G], all quite efficiently.
